# Assessment of Kidney Involvement in COVID-19 Patient

**DOI:** 10.7759/cureus.28964

**Published:** 2022-09-09

**Authors:** Rajkamal Choudhary, Anjum Pervez, Brajesh Kumar, Udit Shankar Singh Patel, Vinay Kumar Verma, Shubham Ojha

**Affiliations:** 1 Department of Medicine, Jawaharlal Nehru Medical College and Hospital, Bhagalpur, IND; 2 Department of Dialysis, Jawaharlal Nehru Medical College and Hospital, Bhagalpur, IND

**Keywords:** polymerase chain reaction, co-relation, chronic kidney disease, covid-19, acute kidney injury

## Abstract

Background and aim: Physicians need to be aware of the difficulties that SARS-CoV-2 infection brings to other regions of the body, such as the kidneys, even though the key emphasis is on pulmonary characteristics. The most frequent kidney complication among COVID-19 hospitalized patients is considered acute kidney injury (AKI). This study aimed to describe overall different aspects of acute kidney injury (AKI) in COVID-19 patients admitted to JLNMCH during the COVID-19 pandemic and to determine the prevalence of AKI among COVID-19 hospitalized patients.

Methods and materials: All adult patients (over the age of 18 years) who screened positive for COVID-19 in a swab specimen from areas of nasopharyngeal by reverse transcriptase polymerase chain reaction and then hospitalized were included in the study. Information was gathered on the patient's demographics, general medical history, and drugs prescribed. From past medical information, associated comorbidities and home pharmaceuticals were identified. We gathered hospitalization information, such as duration of stay in ICU, details about the application of mechanical ventilation, information regarding extracorporeal membrane aeration, details of the use of vasopressor administration, and baseline results of laboratory test along with baseline clinical information during 48 hours of hospitalization.

Results: The percentage of patients with no history of AKI requiring traumatic mechanical ventilation was 79.4%, while the percentage of patients with no history of AKI not requiring traumatic mechanical ventilation was 11.5%. The difference was relevant statistically (p<0.001). The percentage of patients with AKI of any stage requiring traumatic mechanical ventilation was 22.8%, while the percentage of patients with no history of AKI not requiring traumatic mechanical ventilation was 76.8%. The difference was relevant statistically (p<0.022).

Conclusion: We discovered that AKI was a rather typical finding among hospitalized COVID-19 patients. Patients hospitalized for COVID-19 had a poor prognosis if they developed AKI.

## Introduction

The COVID-19 virus has spread globally. The reason there have been so many confirmed cases is that the virus spreads through close contact between people who cough and sneeze while exchanging droplets or aerosols. Engaging on infected surfaces and subsequently touching transmission points like the areas of mouth, areas of eyes, and areas of nose can also result in infection [1,2]. The complete respiratory organ system is the principal organ system affected by COVID-19 infection, which in extreme situations manifests as conditions of pneumonia, hypoxemia, and acute respiratory distress syndrome. Physicians need to be aware of the difficulties that SARS-CoV-2 infection brings to other regions of the body, such as the kidneys, even though the key emphasis is on pulmonary characteristics. The most frequent kidney complication among COVID-19 hospitalized patients is acute kidney injury considered as AKI [3,4].

SARS-CoV-2 is usually an RNA virus that spreads through mouth and nasal secretions, as well as through tiny droplets created by coughing. The reverse transcriptase-polymerase chain reaction (RT-PCR) conducted on respiratory samples acquired by making a swab of the areas of nasopharynx is the usual method of diagnosis. Diverse clinical presentations are possible, including multi-organ failure, mortality, moderate upper respiratory tract disease symptoms, and catastrophic acute breathing distress condition [5,6]. SARS-CoV-2 exploits angiotensin-converting enzyme-2 (ACE-2) to attack numerous organs outside the lungs. One of COVID-19's primary targets in the human body is the kidney. AKI is regarded as a measure of the severity of disease and a poor prognostic variable for patients' survival in COVID-19 conditions [7,8]. Patients who were diagnosed with severe AKI, which was defined as stage 3 or greater in accordance with the recommendations of Kidney Disease Improving Global Outcomes (KDIGO), showed a larger likelihood of passing away than those who were diagnosed with AKI stages 1 and 3 in COVID-19 patients who were impacted by AKI. An increased risk of AKI may occur in individuals who have chronic diseases of the kidney, particularly those suffering from diabetic nephropathy, due to an already present increased regulation of enzyme ACE and reduced regulation of ACE-2 [9].

We saw an incredible amount of patients who experienced AKI, at a significantly higher frequency than those reported from China, when we managed patients suffering from COVID-19. According to early data from China and Italy, the rate of AKI can range between 0.5% and 29%, with many of these estimates falling between these two extremes [10,11].

Clinical data include only critically ill ICU patients showing an overall 19% risk of AKI. Variations could be a consequence of the different populations investigated and the criteria of AKI used. Beyond the rate, there hasn't been much written about AKI in COVID-19; for instance, there aren't many details of the chronology, urine tests, connection to failure of respiratory system, in-depth analyses of the needs for renal replacement therapy for renal replacement, risk variables, or consequences after AKI [12,13].

In this research, we wanted to describe overall different aspects of the experience of AKI in this population of COVID-19 patients and to determine the prevalence of AKI among COVID-19 hospitalized patients.

## Materials and methods

This was a retrospective study conducted at Jawaharlal Nehru Medical College and Hospital during the COVID-19 epidemic, where Jawaharlal Nehru Medical College and Hospital Review Board ethical approval with IRB number JLNMCH/2021/134 was taken. The hospital health record provided the information for this investigation. All adult patients (over the age of 18 years) who screened positive for COVID-19 in a swab specimen from areas of nasopharyngeal by RT-PCR and then hospitalized were included in the study. Transfers of patients among institutions within the same health system were handled as a single hospital engagement.

Patients were disqualified if they had undergone a kidney transplant in the past, had suffered from end-stage renal failure, were relocated to hospitals outside the healthcare system, or had serum levels of serum creatinine that were below 2 at the time of admission. Before the study began, the study protocol was approved by the institutional review board. We obtained preliminary data on AKI as well as renal replacement therapy (RRT) from 8000 COVID-19 patients who died or were discharged from our hospital.

KDIGO guidelines were used to define AKI in the following manner: stage 1 involves an elevation in creatinine level in serum of 0.3 mg/dl during 48 h or even a 1.5-fold to 1.9-fold elevation from values at baseline under seven days, stage 2 involves a 2.9-fold increase under seven days, and stage 3 involves an increment of three-fold or greater within seven days or the start of RRT. Participants were divided into groups based on the maximum AKI stage they had reached while in the hospital.

By using creatinine equation developed by the Chronic Disease of Kidney Epidemiology Consortium, the estimated rate of glomerular filtration was determined. Additionally, we gathered the findings of computerized microscopy urine analysis and urine electrolytes tests that were performed within 24 h or 48 h following the onset of AKI. There was no way for this investigation to determine if a urethral catheter was in place.

Outcomes

The emergence of AKI was the main outcome. Hospital disposition including discharge of the patient from hospital or death and the requirement for RRT were secondary results. In our healthcare system, individuals with AKI could choose between interrupted hemodialysis and continuous CRRT as their RRT options.

Covariates

We gathered information on the patient's demographics, general medical history, and prescribed drugs. From past medical information, associated comorbidities and home pharmaceuticals were identified. We gathered hospitalization information, such as duration of stay in ICU, details about the application of mechanical ventilation, information regarding extracorporeal membrane aeration, details of the use of vasopressor administration, and baseline results of laboratory test along with baseline clinical information during 48 h of hospitalization. Numerous extra ICUs were built in unconventional hospital sections and units as a result of the COVID-19 epidemic. Therefore, an ICU stay was determined as the requirement for intrusive mechanical breathing, the requirement for vasopressor support, the requirement for assistance from an ICU provider, or the requirement for treatment in a designated ICU location.

Statistic evaluation

For continuous variables with a normally distributed distribution, we calculated means and standard deviations (SD) for without normal distribution variables, medians and interquartile ranges (IQR), and for explanatory data, the percentage was used. Fisher's exact tests were used to compare categorical parameters between individuals with AKI and without AKI, and the nonparametric Kruskal-Wallis test was used to evaluate continuous variables. We used the Kruskal-Wallis test to evaluate the clinical traits of individuals with various stages of AKI.

We used logistic regression analysis with accounting for risk factors that were different between individuals who got AKI compared to those who did not in order to determine risk factors related to the onset of AKI. Whenever the level of the risk factor was less than 0.15, variables were added to the models utilizing a stepwise construction process. A p-value of 0.05 or less was regarded as statistically significant for all two-sided statistical tests.

## Results

The mean age of study participants having no AKI was 60.3 ± 0.05 years. The mean age of study participants having AKI of any stage was 70.1 ± 0.13 years. Study participants having AKI stage 1 was 70.8 ± 0.20 years, AKI stage 2 was 71.2 ± 0.31, and AKI stage 3 was 68.1 ± 0.24 years. The percentage of male in study participants having no AKI was 61.3%, having AKI any stage was 64.8%, having AKI stage 1 was 61.1%, having AKI stage 2 was 61.7%, and having AKI stage 3 was 71.5%. The percentage of female in study participants having no AKI was 41.9%, having AKI any stage was 37.4%, having AKI stage 1 was 41.1%, having AKI stage 2 was 42.6% and having AKI stage 3 was 26.8% (Table 1). AKI was significantly associated with old age and the male gender (Table 1).

**Table 1:** Details about the baseline characteristics of study participants AKI: acute kidney injury

Characteristics	Male (%)	Female (%)	Age (mean±SD)	p-Value
No AKI	61.3	41.9	60.3 ± 0.05	˂0.0001
AKI any stage	64.8	37.4	70.1 ± 0.13	
AKI stage 1	61.1	41.1	70.8 ± 0.20	
AKI stage 2	61.7	42.6	71.2± 0.31	
AKI stage 3	71.5	26.8	68.1 ± 0.24	

The percentage of COVID-19 study participants with history of hypertension was as follows: no AKI (51.5%), AKI any stage (65.8%), AKI stage 1 (68.3%), AKI stage 2 (65.2%), and AKI stage 3 (62.6%). The percentage of COVID-19 study participants with history of coronary artery disease was as follows: no AKI (10.0%), AKI any stage (15.5%), AKI stage 1 (15.7%), AKI stage 2 (17.1%), and AKI stage 3 (14.1%). The percentage of COVID-19 study participants with history of heart failure was as follows: no AKI (5.1%), AKI any stage (11.4%), AKI stage 1 (13.1%), AKI stage 2 (11.5%), and AKI stage 3 (8.9%). The percentage of COVID-19 study participants with history of peripheral, vascular disease was as follows: no AKI (2.1%), AKI any stage (4.1%), AKI stage 1 (3.4%), AKI stage 2 (5.0%), and AKI stage 3 (4.4%). The percentage of COVID-19 study participants with history of diabetes was as follows: no AKI (31.2%), AKI any stage (43.7%), AKI stage 1 (41.8%), AKI stage 2 (42.3%), and AKI stage 3 (45.6%). The percentage of COVID-19 study participants with history of HIV was as follows: no AKI (0.9%), AKI any stage (0.7%), AKI stage 1 (0.8%), AKI stage 2 (1.1%), and AKI stage 3 (0.4%). The percentage of COVID-19 study participants with history of chronic obstructive pulmonary disease was as follows: no AKI (4.5%), AKI any stage (8.7%), AKI stage 1 (10.2%), AKI stage 2 (9.3%), and AKI stage 3 (8.2%) (Table 2).

**Table 2:** Details about the comorbidities in study participants HTN: hypertension; HF: heart failure; CAD: coronary artery disease; PVD: peripheral vascular disease; COPD: chronic obstructive pulmonary disease

	HTN (%)	CAD (%)	HF (%)	PVD (%)	Diabetes (%)	HIV (%)	Chronic liver disease (%)	COPD (%)	BMI (kg/m^2^ )	Cancer
No AKI	51.5	10.0	5.1	2.1	31.2	0.9	74.3	4.5	31.5	7.1
AKI any stage	65.8	15.5	11.4	4.1	43.7	0.7	3.4	8.7	31.2	8.1
AKI stage 1	68.3	15.7	13.1	3.4	41.8	0.8	3.2	10.2	31.9	9.2
AKI stage 2	65.2	17.1	11.5	5.0	42.3	1.1	3.1	9.3	31.7	7.1
AKI stage 3	62.6	14.1	8.9	4.4	45.6	0.4	1.6	8.2	31.7	7.0
p-Value	<0.001	<0.001	<0.001	<0.001	<0.001	0.52	3.22	<0.001	0.08	0.24

The values of SCr in mg/dl at the time of admission in different categories of COVID-19 patients were as follows: no AKI (0.97), AKI any stage (1.36), AKI stage 1 (1.38), AKI stage 2 (1.33), and AKI stage 3 (1.32). The values of SCr in mg/dl at the time of discharge in different categories of COVID-19 patients were as follows: no AKI (0.92), AKI any stage (1.52), AKI stage 1 (1.23), AKI stage 2 (1.59), and AKI stage 3 (4.46). The values of peak SCr in mg/dl in different categories of COVID-19 patients were as follows: no AKI (1.20), AKI any stage (2.61), AKI stage 1 (1.72), AKI stage 2 (2.35), and AKI stage 3 (5.42). The values of median SCr in mg/dl in different categories of COVID-19 patients were as follows: no AKI (0.97), AKI any stage (1.31), AKI stage 1 (1.15), AKI stage 2 (1.21), and AKI stage 3 (2.16). The values of eGFR at admission expressed in ml/min/1.73 m^2^ in different categories of COVID-19 patients were as follows: no AKI (83.6), AKI any stage (57.1), AKI stage 1 (55.3), AKI stage 2 (54.2), and AKI stage 3 (63.1). The values of eGFR at discharge was expressed in ml/min/1.73 m^2^ in different categories of COVID-19 patients were as follows: no AKI (95.1), AKI any stage (46.1), AKI stage 1 (70.1), AKI stage 2 (51.0), and AKI stage 3 (15.1) (Table 3).

**Table 3:** Data regarding various parameters for kidney function SCr: serum creatinine; eGFR: estimated glomerular filtration rate

	Admission SCr (mg/dl)	Discharge SCr (mg/dl)	Peak SCr (mg/dl)	Median SCr (mg/dl)	Admission eGFR (ml/min/1.73 m^2^)	Discharge eGFR (ml/min/1.73 m^2^)
No AKI	0.97	0.92	1.20	0.97	83.6	95.1
AKI any stage	1.36	1.52	2.61	1.31	57.1	46.1
AKI stage 1	1.38	1.23	1.72	1.15	55.3	70.1
AKI stage 2	1.33	1.59	2.35	1.21	54.2	51.0
AKI stage 3	1.32	4.46	5.42	2.16	63.1	15.1
p-Value	<0.001	<0.001	<0.001	<0.001	<0.001	<0.001

There was an analysis regarding the need for invasive mechanical ventilation in different categories of COVID -19 patients. The percentage of patients with no history of AKI requiring traumatic mechanical ventilation was 79.4%, while the percentage of patients with no history of AKI not requiring traumatic mechanical ventilation was 11.5%. The difference was statistically relevant (p<0.001). The percentage of patients with AKI of any stage requiring traumatic mechanical ventilation was 22.8%, while the percentage of patients with no history of AKI not requiring traumatic mechanical ventilation was 76.8%. The difference was statistically relevant (p<0.022). The percentage of patients with AKI stage 1 requiring traumatic mechanical ventilation was 16.4%, while the percentage of patients with no history of AKI not requiring traumatic mechanical ventilation was 24.4%. The difference was not statistically relevant (p<0.062). The percentage of patients with AKI stage 2 requiring traumatic mechanical ventilation was 4.3%, while the percentage of patients with no history of AKI not requiring traumatic mechanical ventilation was 22.0%. The difference was statistically relevant (p<0.052). The percentage of patients with AKI stage 3 requiring traumatic mechanical ventilation was 2.7%, while the percentage of patients with no history of AKI not requiring traumatic mechanical ventilation was 44.6%. The difference was statistically relevant (p<0.001). The percentage of patients with renal replacement therapy requiring traumatic mechanical ventilation was 0.3%, while the percentage of patients with no history of AKI not requiring traumatic mechanical ventilation was 24.3%. The difference was statistically relevant (p<0.001) (Table 4).

**Table 4:** The proportion of patients with AKI by requirement for traumatic mechanical ventilation AKI: acute kidney injury

	No AKI (%)	AKI any stage (%)	AKI stage 1 (%)	AKI stage 2 (%)	AKI stage 3 (%)	Required renal replacement therapy (%)
Traumatic mechanical ventilation was not required	79.4	22.8	16.4	4.3	2.7	0.3
Traumatic mechanical ventilation was required	11.5	76.8	24.4	22.0	44.6	24.3
p-Value	<0.001	<0.022	<0.062	<0.052	<0.001	<0.001

When there was analysis regarding factors associated with AKI in patients with COVID-19 then it was found that factors like old age, male gender, history of diabetes, hypertension, cardiovascular disease, mechanical ventilation, use of vasoactive medication, and use of angiotensin-converting enzyme-1 (ACE-1) or angiotensin receptor blocker (ARB) were associated with AKI significantly (Table 5).

**Table 5:** Univariate and multivariate logistic regression analyses of risk factors associated with the development of AKI AKI: acute kidney injury; ACE-1: angiotensin-converting enzyme-1; ARB: angiotensin receptor blocker

	Diabetes	Hypertension	Cardiovascular disease	Mechanical ventilation	Vasoactive medication	ACE-1 or ARB use
Unadjusted OR	1.83	1.88	2.05	31.60	32.51	1.71
95% CI	1.62-2.07	1.62-2.13	1.87-2.47	25.80-38.60	25.71-39.40	1.52-1.92
p-Value	<0.021	<0.062	<0.034	<0.013	<0.033	<0.002
Adjusted OR	1.78	1.46	1.48	11.8	4.64	0.98
95% CI	1.50-2.18	1.16-1.61	1.22-1.80	6.92-16.81	2.89-7.23	0.74-1.15
p-Value	<0.001	0.03	<0.001	<0.001	<0.001	0.01

## Discussion

When we treated COVID-19 patients, we observed a staggering number of patients who had AKI, at a frequency that was noticeably higher than those reported from China. The rate of AKI can range from 0.5% to 29%, with many of these estimates falling between these two extremes, according to early data from China and Italy. Only critically sick ICU patients are included in clinical data, and the total risk of AKI is 19%. Variations may result from the many populations examined and the various AKI criteria applied [14,15]. Except for the rate, not much has been written about AKI in COVID-19; for example, there are few details about the chronology, urine tests, connection to respiratory system failure, in-depth analyses of the needs for renal replacement therapy for renal replacement, risk factors, or consequences after AKI [16,17].

We aimed to find out the prevalence of AKI among hospitalized COVID-19 patients and describe the various general characteristics of AKI experience in this cohort of patients. KDIGO guidelines were used to define AKI in the following manner: stage 1 involves an elevation in creatinine level in serum of 0.3 mg/dl during 48 h or even a 1.5-fold to 1.9-fold elevation from values at baseline under seven days, stage 2 involves a 2.9-fold increase under seven days, and stage 3 involves an increment of three-fold or greater within seven days or the start of RRT. Participants were divided into groups based on the maximum AKI stage they had reached while in the hospital.

Thirty-four patients with AKI/CKD were admitted to JLNMCH, Bhagalpur, during the COVID-19 pandemic. Out of 8000 patients with COVID-19 admitted in JLNMCH, 460 patients were admitted to ICU of which we had 34 patients who went for renal dialysis. Seven patients were of AKI and the rest was CKD. In patients with AKI, serum creatinine levels were found between seven and 16, while in patients with CKD, the level was between five and 12. Patients usually had a history of diabetes and hypertension in CKD while anuria was the major complaint of AKI. Hemoglobin level was markedly reduced in patients with CKD.

When there was analysis regarding factors associated with AKI in patients with COVID-19 then it was found that factors like old age, male gender, history of diabetes, hypertension, cardiovascular disease, mechanical ventilation, use of vasoactive medication and use of ACE-1 or ARB were associated with AKI significantly.

In this study, the mean age of participants having no AKI was 60.3 ± 0.05 years, participants having AKI of any stage was 70.1 ± 0.13 years, participants having AKI stage 1 was 70.8 ± 0.20 years, participants having AKI stage 2 was 71.2± 0.31, and participants having AKI stage 3 was 68.1 ± 0.24 years. The percentage of male study participants having no AKI was 61.3%, having AKI of any stage was 64.8%, having AKI stage 1 was 61.1%, having AKI stage 2 was 61.7%, and having AKI stage 3 was 71.5%. The percentage of female study participants having no AKI was 41.9%, having AKI any stage was 37.4%, having AKI stage 1 was 41.1%, having AKI stage 2 was 42.6%, and having AKI stage 3 was 26.8% (Table 1). AKI was significantly associated with old age and male gender.

In this study, there was an analysis regarding the need for invasive mechanical ventilation in different categories of COVID-19 patients. The percentage of patients with no history of AKI requiring traumatic mechanical ventilation was 79.4%, while the percentage of patients with no history of AKI not requiring traumatic mechanical ventilation was 11.5%. The difference was relevant statistically (p<0.001). The percentage of patients with AKI of any stage requiring traumatic mechanical ventilation was 22.8%, while the percentage of patients with no history of AKI not requiring traumatic mechanical ventilation was 76.8%. The difference was relevant statistically (p<0.022).

SARS-CoV-2 is typically an RNA virus that spreads by secretions from the mouth and nose as well as via microscopic droplets produced while coughing. The standard procedure for diagnosis uses RT-PCR on respiratory samples obtained by taking a swab from the nasopharynx. Multiple organ failure, death, mild upper respiratory tract disease symptoms, and severe acute breathing distress are just a few examples of the various clinical presentations that could occur [18,19]. SARS-CoV-2 attacks a variety of organs outside the lungs by taking advantage of the angiotensin-converting enzyme-2 (ACE-2). The kidney is one of COVID-19's main targets in the human body. In the COVID-19 illness, AKI is viewed as a marker of disease severity and a poor predictor of patient survival [20,21]. According to KDIGO recommendations, patients with severe AKI defined as stage 3 or above showed a higher death rate than those with AKI stage 1 and AKI stage 3 in COVID-19 afflicted individuals with AKI. Due to an already existing elevated regulation of enzyme ACE and reduced regulation of enzyme ACE-2, patients with chronic illnesses of the kidney, particularly those suffering from diabetic nephropathy, may be at an increased risk of developing AKI [22,23].

Participants in the COVID-19 research who had a history of hypertension were more likely to have no AKI (51.5%), any stage of AKI (65.8%), stage 1 AKI (68.3%), stage 2 AKI (65.2%), and stage 3 AKI (62.6%). The percentage of COVID-19 study participants with history of coronary artery disease was as follows: no AKI (10.0%), AKI any stage (15.5%), AKI stage 1 (15.7%), AKI stage 2 (17.1%), and AKI stage 3 (14.1%). The percentage of COVID-19 study participants with history of heart failure was as follows: no AKI (5.1%), AKI any stage (11.4%), AKI stage 1 (13.1%), AKI stage 2 (11.5%), and AKI stage 3 (8.9%).

Every nation now has the COVID-19 virus. The virus spreads through close contact between people who cough, sneeze, or speak while exchanging droplets or aerosols, which is why there have been so many confirmed cases. Infection can also occur by interacting with infected surfaces and then touching transmission points like the mouth, eyes, and nose. The primary organ system impacted by COVID-19 infection is the respiratory system as a whole. In severe cases, this infection can cause pneumonia, hypoxemia, and acute respiratory distress syndrome [24,25]. Even though the pulmonary characteristics of SARS-CoV-2 infection are the main focus, doctors need to be mindful of the challenges that illness brings to other parts of the body, such as the kidneys. Acute kidney injury (AKI), often known as AKI, is the kidney symptom that hospitalized COVID-19 patients experience the most frequently [26].

Our study offers some advantages. This cohort of COVID-19 patients that are hospitalized and have an emphasis on AKI is now by far the largest. There are restrictions on this study. First, because it is observational research, we are unable to draw conclusions about the causal links between various levels of exposure to AKI. Second, despite the fact that we have made adjustments for possible confounders, there can still be unmeasured confounders.

## Conclusions

In conclusion, we discovered that AKI was a rather typical finding among hospitalized COVID-19 patients. It was a rare condition when COVID-19 individuals did not need ventilation and were closely related to the incidence of respiratory failure. Hospitalized COVID-19 patients had a poor prognosis if they developed AKI. To further comprehend the causes of AKI including patient outcomes, more research will be required.
